# Antibiotic-resistant bacteria in hospital wastewater treatment plant effluent and the possible consequences of its reuse in agricultural irrigation

**DOI:** 10.3389/fmicb.2023.1141383

**Published:** 2023-04-18

**Authors:** Magda M. Mehanni, Samir I. Gadow, Fahdah Ayed Alshammari, Yosra Modafer, Kholoud Z. Ghanem, Noha Fathy El-Tahtawi, Rania F. El-Homosy, Abd El-Latif Hesham

**Affiliations:** ^1^Department of Botany and Microbiology, Faculty of Science, Minia University, Minya, Egypt; ^2^Department of Agricultural Microbiology, Agriculture and Biology Research Institute, National Research Centre, Cairo, Egypt; ^3^Department of Biology, Faculty of Science and Arts-RAFHA, Northrn Border University, Arar, Saudi Arabia; ^4^Department of Biology, College of Science, Jazan University, Jazan, Saudi Arabia; ^5^Department of Biological Sciences, College of Science and Humanities, Shaqra University, Shaqra, Saudi Arabia; ^6^Department of Biology, College of Science and Arts, Shaqra University, Shaqra, Saudi Arabia; ^7^Department of Genetics, Faculty of Agriculture, Assiut University, Assiut, Egypt; ^8^Genetics Department, Faculty of Agriculture, Beni-Suef University, Beni-Suef, Egypt

**Keywords:** Hospital wastewater treatment plant, multi-drug resistant bacteria, 16S rRNA gene sequencing, antibiotic resistance profiles, greenhouse experiment

## Abstract

Wastewater from hospitals should be monitored precisely and treated properly before discharge and reuse to avoid epidemic and pandemic complications, as it contains hazardous pollutants for the ecosystem. Antibiotic residues in treated hospital wastewater effluents constitute a major environmental concern since they resist various wastewater treatment processes. The emergence and spread of multi-drug-resistant bacteria, that cause public health problems, are therefore always a major concern. The aims and objectives of this study were mainly to characterize the chemical and microbial properties of the hospital effluent of wastewater treatment plant (WWTP) before discharge to the environment. Special attention was paid to the presence of multiple resistant bacteria and the effects of hospital effluent reuse in irrigation on zucchini as an economically important plant. The risk of cell-free DNA carrying antibiotic resistance genes contained in the hospital effluent as a long-lasting hazard had been discussed. In this study, 21 bacterial strains were isolated from the effluent of a hospital WWTP. Isolated bacteria were evaluated for multi-drug resistance ability against 5 antibiotics (Tetracycline, Ampicillin, Amoxicillin, Chloramphenicol, and Erythromycin) at a concentration of 25 ppm. Out of them, three isolates (AH-03, AH-07, and AH-13) were selected because they recorded the highest growth in presence of tested antibiotics. Selected isolates were identified using 16S rRNA gene sequence homology as *Staphylococcus haemolyticus* (AH-03), *Enterococcus faecalis* (AH-07), and *Escherichia coli* (AH-13). Their susceptibility to ascending concentrations of tested antibiotics indicated that they were all susceptible at a concentration above 50 ppm. Results of the greenhouse experiment regarding the effect of hospital WWTP effluent reuse on zucchini plant fresh weights compared to that irrigated with fresh water indicated that the former recorded a limited increase in total fresh weights (6.2 g and 5.3 g/plant, respectively). Our results demonstrated the low impact of the reuse of Hospital WWTP effluent in agriculture irrigation compared to its greater risk in transferring multiple antibiotic bacteria and antibiotic resistance genes to soil bacteria through natural transformation.

## 1. Introduction

Hospitals are considered as one of the most polluting sectors around the world (Achak et al., 2021). Wastewater treatment plants (Hospital wastewater treatment plants in particular) are also considered and best described as hot spots for the dissemination of antibiotic resistance that could threat public health upon water reuse (Rizzo et al., 2013; Yuan and Pia, 2023). The biological treatment of wastewater with microorganisms removes contaminants (e.g., organic carbon, nutrients, and micropollutants). Each treatment system has its microbial community structure that evolves and is composed of a variety of microorganisms. The abundance of antibiotic resistance genes (ARGs) in the environment has become a major global public health concern as a result of the widespread use of antibiotics in healthcare systems, agriculture, and breeding (Ju et al., 2016; Ma et al., 2017). Previous research has discovered significant amounts of antibiotics and residual bacteria in hospital wastewater (HWW), which can act as a selective pressure on antibiotic-resistant bacteria growth (Rowe et al., 2017). As a result, HWWs are more likely than other wastewater systems, such as urban wastewater systems, to spread ARGs (Verlicchi et al., 2015; Zheng et al., 2018). Glycopeptides, carbapenems, and other antibiotics are used more frequently in hospitals than in other settings. As a result, solid waste ARG profiles differ from those of other sewage systems (Kümmerer and Henninger, 2003). This distinction raises the prospect of hospital-related ARGs becoming more common. Since the 1980s, regulations for sludge/wastewater emission standards have been established all over the world to reduce the harm caused by post-discharge effluent (Meng et al., 2016). However, only a few countries (for example, France and Italy) have made pre-release HWWs treatment legal (Boillot et al., 2008; Verlicchi et al., 2010; Al Aukidy et al., 2017). Unfortunately, ARG biological safety is not a mandatory criterion for wastewater emissions (Guo et al., 2016). The current situation suggests that ARGs from HWW pose a significant biosafety risk, which has gone largely unnoticed in wastewater treatment facilities and regulations (Guo et al., 2016). Furthermore, after treatment and unloading, ARGs are continuously propagated within the microbial community *via* horizontal gene transfer (Barancheshme and Munir, 2017; Kümmerer et al., 2018). Because of differences in antibiotic application patterns, antibiotic resistance levels in hospital wastewater may differ from those found in other aquatic environments. Certain antibiotics, such as cefotiam, piperacillin, and vancomycin, are mostly used in hospitals (Kümmerer and Henninger, 2003). Increasing population and fixed water supply result in less water per person yearly. United nation predicts that by 2025, Egypt will reach an absolute water crisis due to the country’s low level of water poverty. As a result, the goal of this research was to isolate and characterize multi-antibiotic-resistant bacteria from hospital effluent of WWTP to determine the threat from bacterial genera that persist after treatment and their resistance pattern to some of the commonly used antibiotics. Also, exploring the effect of hospital effluent water on plant growth was another goal of this study in case agricultural soils were an effluent receiving environment. In that case, the removal of free DNA from hospital effluent water is extremely important to prevent the transfer of antibiotic resistance gene to soil bacteria which, if happened, can lead to a public massive health crisis.

## 2. Materials and methods

### 2.1. Sample collection

Wastewater samples were collected from Beni-Suef city at different intervals as the following:

(1) From the untreated wastewater outlet pipe of a selected hospital (Beni-Suef university hospital) before it enters the sewer system, (2) Sewage treatment plant (using activated sludge), and (3) Treated water (effluent) before being discharged. A volume of 2 liters of wastewater was collected from each site using new first use sterile plastic bottles (sterilized by shaking with 70% ethanol for 3 min followed by three times rinsing with sterile distilled water), preserved on ice, and transported to the laboratory for physical, chemical, and microbiological analyses. Also, Fresh sludge samples were obtained from the WWTP for analysis.

### 2.2. Analysis of physical, chemical, and microbiological parameters for collected hospital wastewater samples

Different physical and chemical parameters of collected raw hospital wastewater samples were determined according to American Public Health Association (APHA) standard methods (Rice et al., 2012; Baird and Bridgewater, 2017). These parameters include pH, biochemical oxygen demand (BOD), total chemical oxygen demand (TCOD), volatile suspended solid (VSS), total suspended solids (TSS), volatile solid (*VS*), and total solid (TS).

Microbiological parameters were determined for the effluent of HWWTP, as shown in Table 1. Dilutions of wastewater samples were performed and varied from 10^−1^ to 10^−7^. For pathogenic bacteria characterization, MacConkey broth without crystal violet, that is a differential medium less selective than MacConkey medium, so it permits growth of enterococci, staphylococci, and *Mycobacterium* spp., was used. Dehydrated MacConkey broth was bought from HiMedia (India) and prepared according to the manufacturer’s instructions, then distributed into a screw-capped bottle fitted with an inverted Durham tube for the determination of coliform bacteria (total coliforms, TC, and fecal coliforms, FC) by most probable number method (Rice et al., 2012). Sterilization is done by autoclaving at 121°C for 15 min under a pressure of 15 lb. per square inch. The colony-forming unit (CFU), Fungi, and Yeast were determined based on the plate-counting method using specific media as in APHA (Young et al., 2005).

**Table 1:** Microbiological characterization of the effluent quality.

	CFU	Fungi	Pseudomonas spp.	YPD (Yeast)	Total coliforms, TC (MPN/100 mL)	Fecal coliforms, FC (MPN/100 mL)
Raw (effluent) wastewater	67 × 10^7^	7 × 10^7^	160 × 10^8^	155 × 10^8^	5.4 × 10^8^	2.4 × 10^6^

### 2.3. Bacterial isolation from treated hospital wastewater effluent

Serial 10-fold dilutions of wastewater samples were prepared, and 0.1 mL aliquots were inoculated onto MacConkey agar without crystal violet (for *Escherichia coli and Enterococcus* spp.) and on nutrient agar culture media, then plates were incubated aerobically at 30°C for 24 h. Colonies with different morphologies were recovered from each plate, streaked on the same isolation medium to obtain pure cultures. Isolates were maintained at 4°C as agar slants and as glycerol stocks at −20°C in the same media broth containing 25% glycerol (Rice et al., 2012).

### 2.4. Screening for multiple antibiotic-resistant bacteria

All isolated bacteria were evaluated for antibiotic-resistant using nutrient broth culture media supplemented individually with 5 different, filter-sterilized, antibiotic solutions (Tetracycline, Ampicillin, Amoxicillin, Chloramphenicol, and Erythromycin) at a concentration of 25 ppm. Flasks were incubated for 24 h at 30°C on a rotary shaker at 200 rpm. The growth of the bacterial isolates was measured by recording the OD readings at 600 nm against nutrient medium broth as blank. Isolates showing the highest growth during screening as demonstrated by the increase in their optical densities (OD600) were selected for further studies.

### 2.5. Molecular identification of isolated bacteria

Selected bacterial isolates were identified at the species level based on the analysis of their 16S ribosomal RNA gene sequences.

#### 2.5.1. DNA extraction and PCR amplification of 16S rRNA gene

The total genomic DNA from the selected bacteria was extracted according to the method described by Hesham (2014). DNA was amplified by PCR using 16S rRNA universal primers 27F (5-AGAGTTTGATCCTGGCTCAG-3) and 1492R (5-CGGCTACCTTGTTACGACTT-3) (Lane, 1991). The PCR reaction was performed in 50 μl as a final volume as described in Hesham, 2014. Five microliter of the amplified mixture was then analyzed using agarose gel electrophoresis (1% agarose and 0.5 × TBE). The gel was stained with ethidium bromide, visualized under UV light, and photographed.

#### 2.5.2. PCR products purification and sequence determination

To verify the presence of appropriate-sized amplicons, the PCR product for each selected bacteria was subjected to electrophoresis in 1% agarose gel according to standard methods. The product of the correct size was purified with a TaKaRa Agarose Gel DNA Purification Kit version 2.0 and sequenced in both directions using an ABI 3730 automated sequencer (Macrogen, Seoul, Korea).

#### 2.5.3. Comparison of 16S rRNA gene sequences with GenBank database

The obtained data of 16S rRNA gene sequences were aligned and compared with those available in the GenBank database as previously described in Hesham et al. (2016).

#### 2.5.4. Phylogenetic analysis

To determine the taxonomic position of the selected bacteria, phylogenetic trees were constructed with MEGA version 4.0 using a neighbor-joining algorithm, and the Jukes-Cantor distance estimation method with bootstrap analyses for 1,000 replicates.

### 2.6. Determination of resistance profiles against the 5 tested antibiotics

Antimicrobial resistance for the selected bacteria to ascending concentrations of the previously tested antibiotics at ~25 ppm (Tetracycline, Ampicillin, Amoxicillin, Chloramphenicol, and Erythromycin) was performed at a range from 5 to 250 ppm (5, 10, 15, 25, 50, 150, 200, and 250 ppm). Filter sterile antibiotic solutions were added separately to nutrient broth media to achieve the desired concentrations. Flasks were incubated for 24 at 30°C on a rotary shaker at 200 rpm. The growth of the bacterial strains was measured by recording the OD at 600 nm against nutrient medium broth as blank.

### 2.7. Pots experiment setup

A series of pot experiments were conducted in the greenhouse conditions at National Research Centre, Egypt, using the commercial zucchini plant (H5N5) to test the effect of hospital wastewater treatment plant (WWTP) effluent reuse in irrigation on zucchini seedlings growth compared to those irrigated with fresh water. Soil physical properties were analyzed using the procedures described by Black et al. (1981). While soil chemical analysis was measured according to the procedures described by Jackson (1973). The microbiological characterizations were performed according to methods described in APHA (2001).

### 2.8. GenBank nucleotide sequences submission

The nucleotide sequences of 16S rRNA gene of the strains AH-03, AH-07, and AH-13, isolated in this study have been deposited in the GenBank^1^ under the accession numbers ON873252, ON873253, and ON873254, respectively.

### 2.9. Statistical analysis

Analysis of variance (ANOVA) was carried out to detect the significance of differences between the treatment means (Gomez and Gomez, 1984).

## 3. Results

### 3.1. Raw hospital wastewater characterization

The parameters of BOD5 and COD are widely used to characterize the organic matter of wastewater. The minimum concentrations of BOD5 and COD were 150.1 and 381.2 mg/L, while the maximum levels were 180.2 and 431.1 mg/L. One of the common parameters used in defining wastewater is TSS. The average TSS in this study was 229 mg/L with turbidly reaching 60 NTU.

### 3.2. Isolation of bacteria from the effluent of the hospital wastewater treatment plant

A total of 21 bacterial isolates were recovered on Nutrient agar (all-purpose culture medium) and MacConkey agar medium without crystal violet (selective culture medium).

### 3.3. Screening isolated bacteria for multiple antibiotic resistance

All of the 21 bacterial isolates were individually evaluated for their resistance against the 5 tested antibiotics at a concentration of 25 ppm each. Results indicated that three isolates (AH-03, AH-07, and AH-13) showed the highest growth among other isolates under the selective pressure of 25 ppm concentration of tested antibiotics as they recorded the highest growth that expressed as the increase in their optical densities at OD600.

### 3.4. 16S rRNA gene-based identification of isolated bacteria

**Figure 1:**
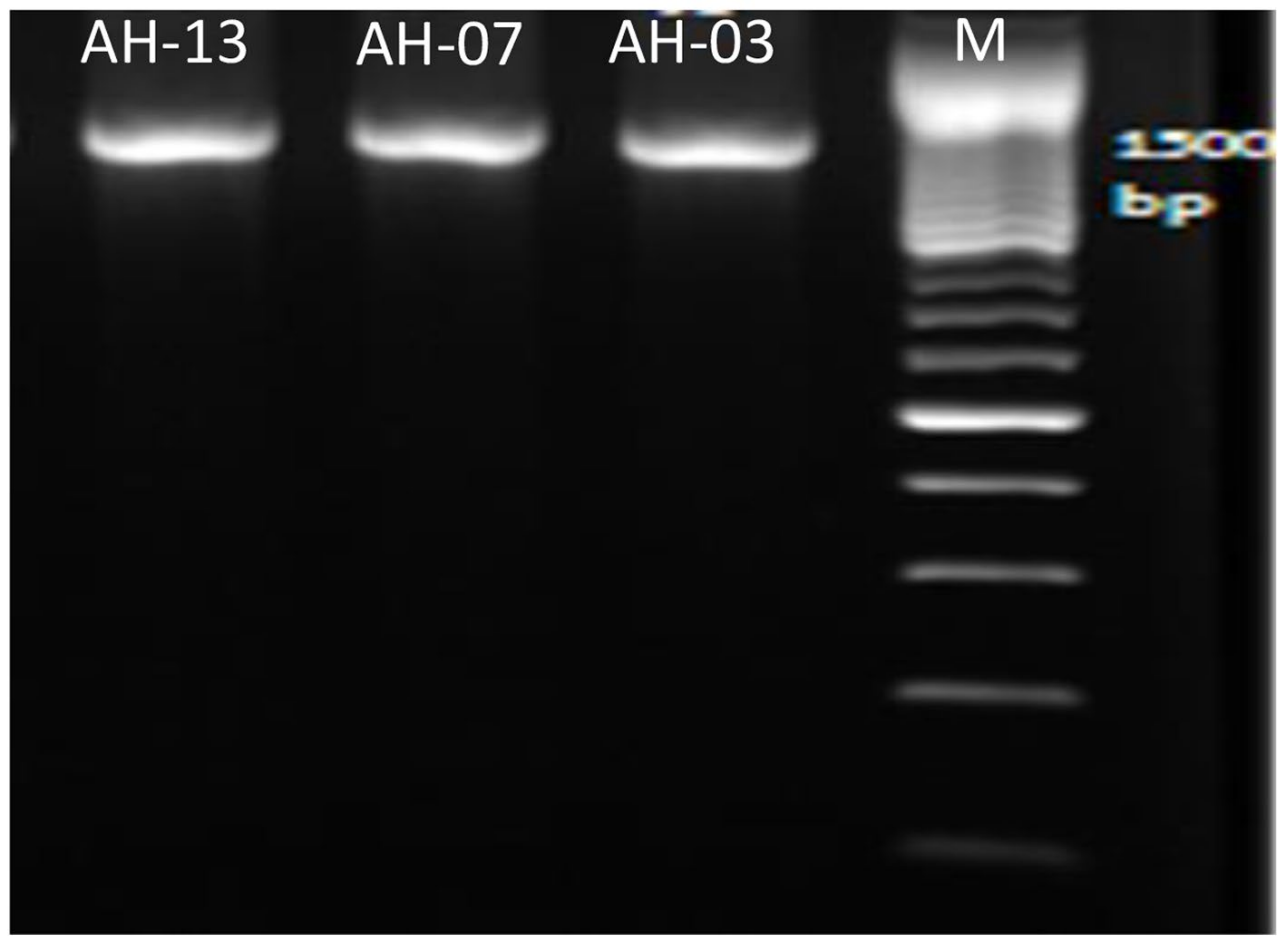
Amplified DNA of the 16S rRNA gene for the selected isolates with primer pair 27F and 1492R, Lane 1: 100 bp DNA markers; Lane 2: strain AH-03; Lane 3: strain AH-07 and Lane 4: strain AH-13.

To identify and determine the correct phylogenetic position of the selected isolates, Amplification and sequencing of their16S rRNA region was performed. All of the three selected isolates were shown to have PCR amplified fragments with around 1,500 bp (Figure 1).

Alignment of the 16S rRNA gene sequences of these isolates with sequences obtained from BLAST search revealed up to 100% identity to different bacteria. Strains AH-03, AH-07, and AH-13 demonstrated 100% identity to *Staphylococcus haemolyticus, Enterococcus faecalis,* and *Escherichia coli*, respectively.

### 3.5. Construction and analysis of phylogenetic trees for isolated bacteria

**Figure 2:**
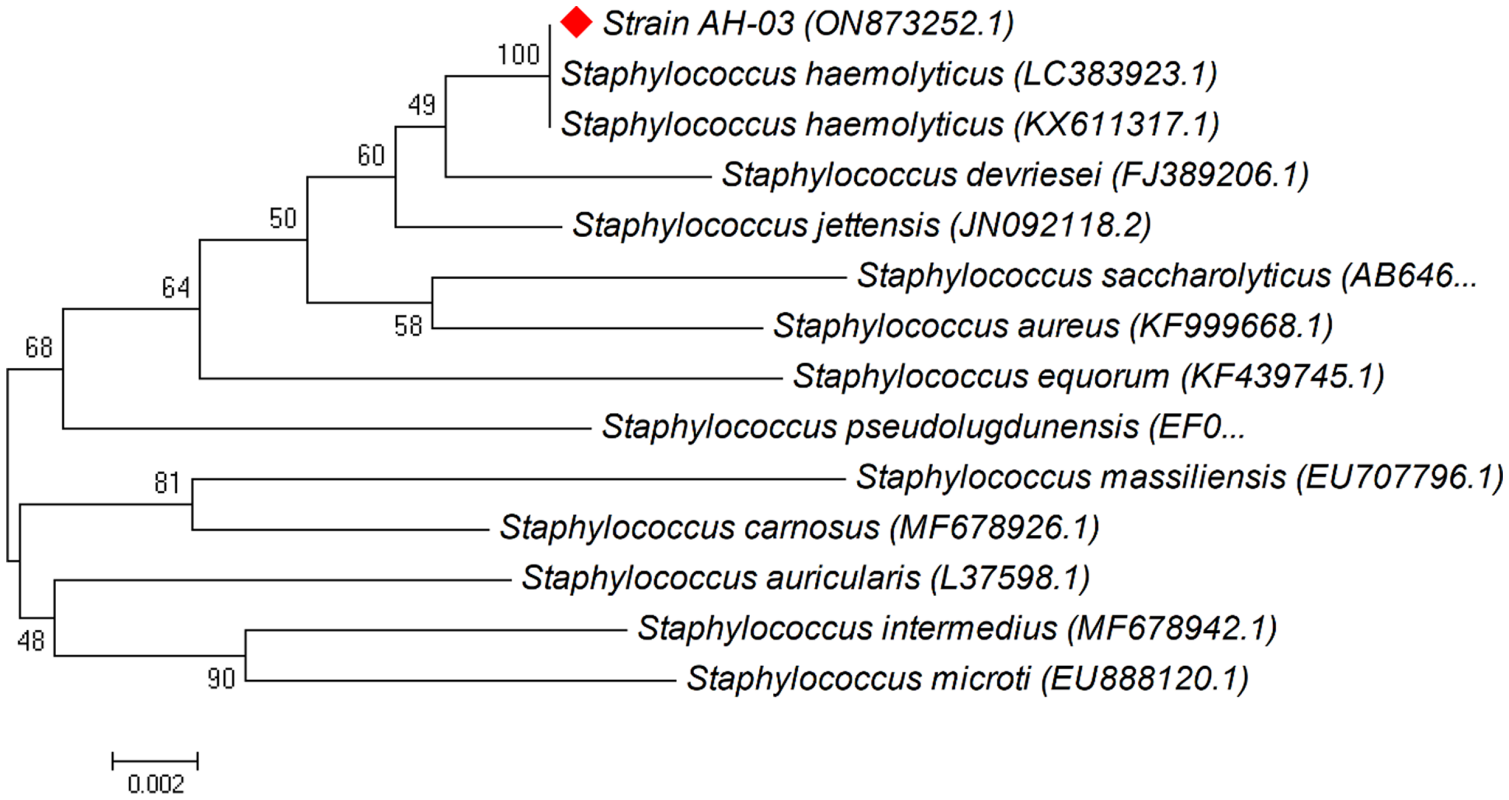
Phylogenetic relationship between the strain AH-03 and other 16S rRNA gene sequences of published strains. In the phylogenetic tree, AH-03 and *Staphylococcus haemolyticus* were clustered together as one clade. The scale bar corresponds to a 0.002 nucleotide substitution per sequence position. The number in parentheses represents the accession number in GenBank.

**Figure 3:**
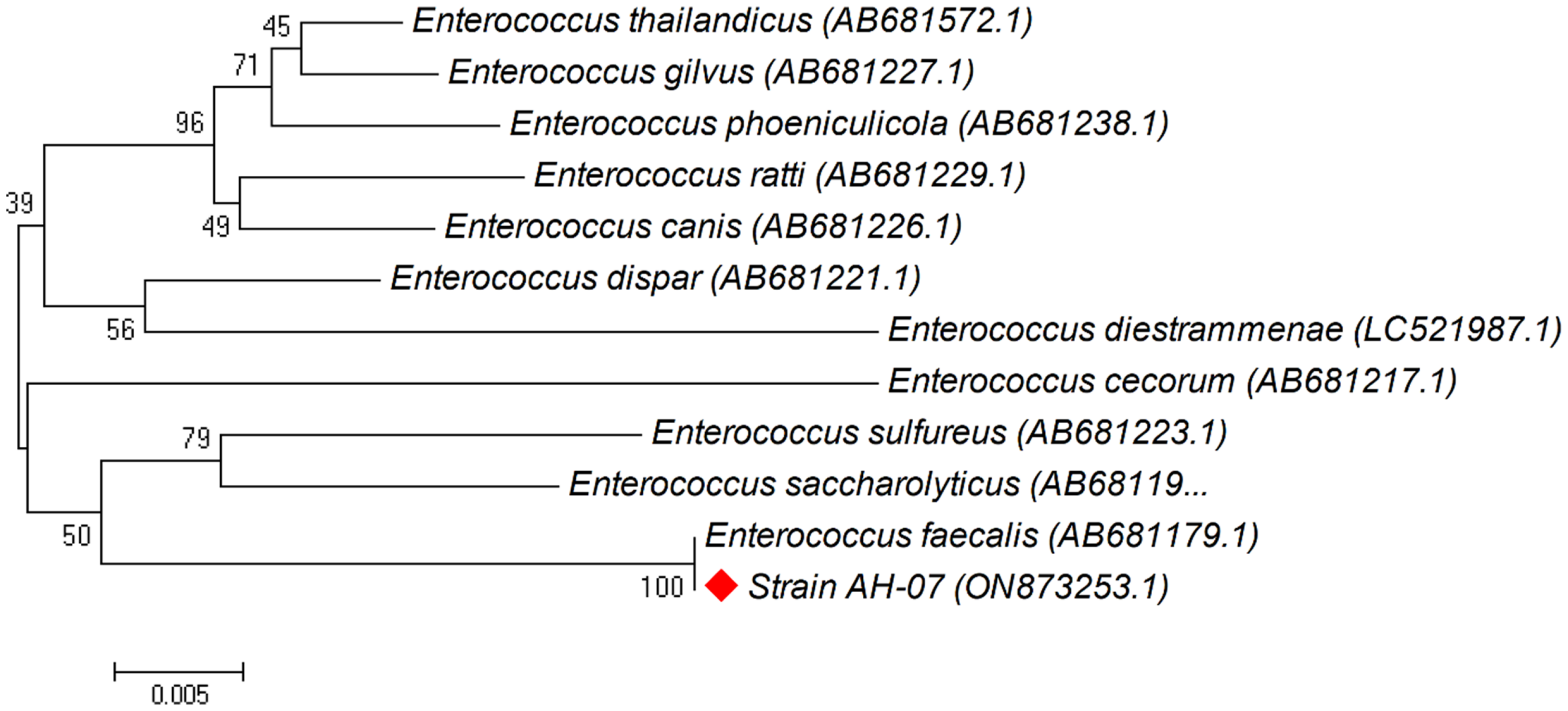
Phylogenetic relationship between the strain AH-07 and other 16S rRNA gene sequences of published strains. In the phylogenetic tree, AH-07 and *Enterococcus faecalis* were clustered together as one clade. The scale bar corresponds to a 0.005 nucleotide substitution per sequence position. The number in parentheses represents the accession number in GenBank.

**Figure 4:**
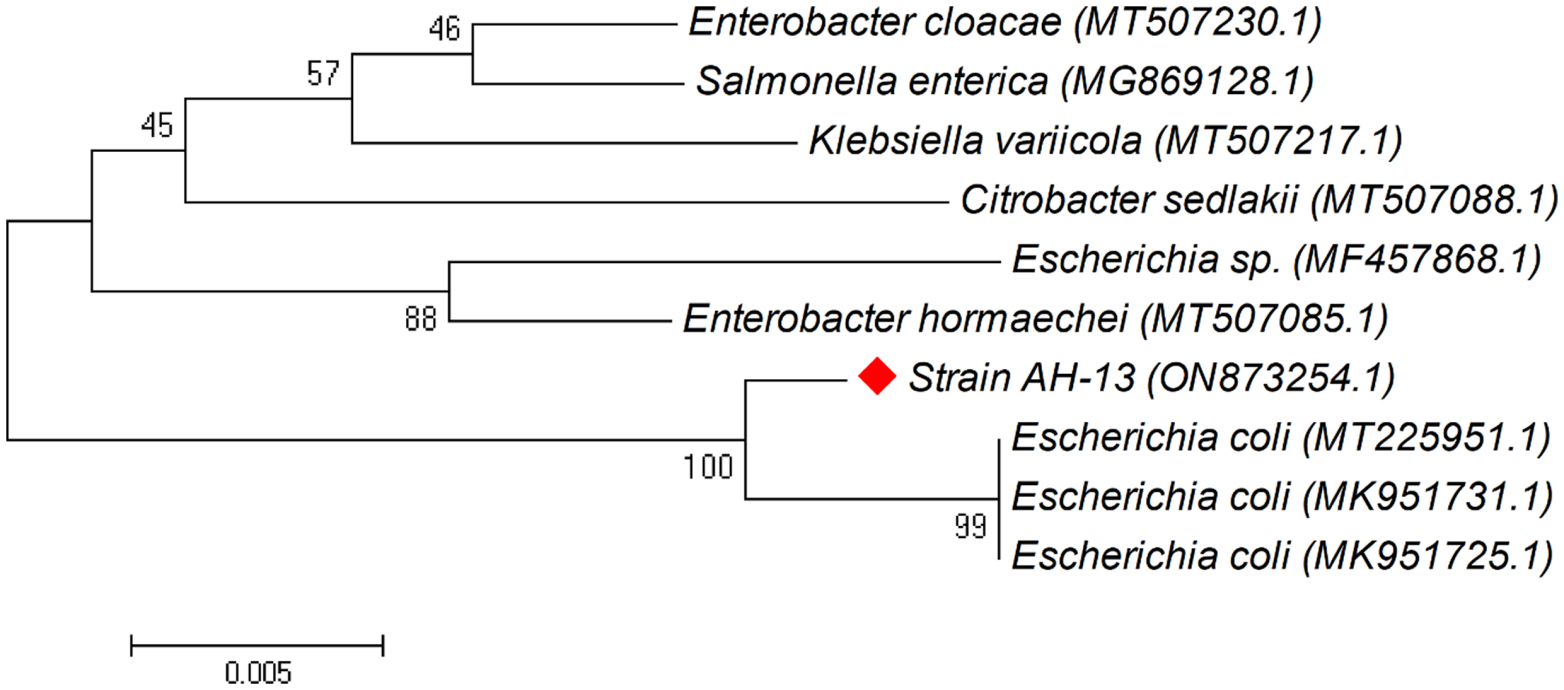
Phylogenetic relationship between the strain AH-13 and other 16S rRNA gene sequences of published strains. In the phylogenetic tree, AH-13 and *Escherichia coli* were clustered together as one clade. The scale bar corresponds to a 0.005 nucleotide substitution per sequence position. The number in parentheses represents the accession number in GenBank.

Phylogenetic trees were constructed between the pairwise 16S rDNA sequences of isolated strains and the closely similar homologs. The phylogenetic tree analysis indicated that strain AH-03 and *S. haemolyticus* shared one clade cluster (Figure 2), Therefore, strain AH-03 was identified as a strain of *S. haemolyticus*. Isolate AH-07 was identified as *E. faecalis* as they shared one clade cluster (Figure 3). AH-13 isolate was identified as a strain of *E. cloi* since they shared one clade cluster in the constructed phylogenetic tree (Figure 4).

### 3.6. Antibiotic resistance profiles for isolated bacteria

Resistance of isolated bacterial strains, *S. haemolyticus* AH-03, *E. faecalis* AH-07, and *E. coli* AH-13 against ascending concentrations of tested antibiotics, indicated that the three isolates were resistant to the 5 tested antibiotics at concentrations up to 25 ppm, while they were all susceptible at a concentration above 50 ppm (Table 2).

**Table 2:** Antibiotic resistance profiles for the selected isolates AH-03, AH-07, and AH-13.

Strains	*Escherichia coli*					*Enterococcus faecalis*					*Staphylococcus haemolyticus*				
Antibiotic Conc. (ppm)	Tet	Amp	Amox	Chl	Er	Tet	Amp	Amox	Chl	Er	Tet	Amp	Amox	Chl	Er
5	R	R	R	R	R	R	R	R	R	R	R	R	R	R	R
10	R	R	R	R	R	R	R	R	R	R	R	R	R	R	R
15	R	R	R	R	R	R	R	R	R	R	R	R	R	R	R
25	R	R	R	MS	MS	R	R	MS	R	R	R	R	MS	R	MS
50	MS	MS	MS	S	s	MS	MS	S	S	MS	MS	MS	S	MS	s
75	S	S	S	S	S	S	S	S	S	S	S	S	S	S	S
150	S	S	S	S	S	S	S	S	S	S	S	S	S	S	S
200	S	S	S	S	S	S	S	S	S	S	S	S	S	S	S
250	S	S	S	S	S	S	S	S	S	S	S	S	S	S	S

R, Resistance; MS, Medium Susceptible; S, Susceptible (After 24 h incubation). Tetracycline (Tet), Ampicillin (Amp), Amoxicillin (Amox), Chloramphenicol (Chl) and Erythromycin (Er).

### 3.7. Effect of hospital WWTP effluent on zucchini growth

Experimental results indicated that the mean values for total fresh weight per plant were 5.3 and 6.2 for plants irrigated with fresh water and treated hospital wastewater, respectively. The mass production of both roots and leaves of the latter was consequently improved (Table 3).

**Table 3:** Effect of irrigation quality on zucchini production.

Treatments	Total weight / plant (g)	Root weight/plant (g)	Leaves weight/plant (g)
FW	5.18 ± 0.16	0.13 ± 0.01	2.83 ± 0.05
TW	6.18 ± 0.12	0.48 ± 0.03	2.23 ± 0.01
UTW	4.80 ± 0.0	0.24 ± 0.01	2.43 ± 0.13

Each value is the average of three replicates ± standard error. FW, Fresh Water; TW, Treated Water at 6 HRT; UTW, Untreated Wastewater.

## 4. Discussion

Wastewaters from hospitals contain considerable amounts of chemicals, microbial agents, and cell-free DNA. Chemicals present in hospital wastewater belong to different groups and many of them resist normal wastewater treatment. They end up in surface waters where they can affect the aquatic ecosystem. Humans are particularly exposed to drinking water, produced from surface water (Pauwels and Verstraete, 2006).

In most hospitals, the BOD5 and COD concentrations of wastewater are almost equal to domestic wastewater values. In another study, the average of BOD5 and COD in wastewaters of Teheran hospitals was 444.3 mg/L and 792 mg/L, respectively (Tchobanoglous et al., 2004). The results indicated that the COD removal reached 92.4%. While growing efficiency can be significantly influenced by environmental conditions, the ratio COD/BOD must be considered as a function of growth efficiency. The high biodegradability of organic matter is very desirable from the viewpoint of wastewater treatment and promotes the efficiency of wastewater treatment plants (Mesdaghinia et al., 2009). One of the common parameters used in defining wastewater is TSS. The average TSS in this study was 229 mg/L with turbidly reaching 60 NTU. Moersidik studied the wastewater quality of a hospital in Indonesia and found TSS concentration to range from 36 to 269 mg/L. Hospital wastewater effluents contain pathogenic microorganisms, pharmaceuticals partially metabolized, radioactive elements, heavy metals, and toxic chemicals (Moersidik, 1993; Brown et al., 2006).

The residual antibiotics can reach the water environment through wastewater (Heberer, 2002) and they can induce bacterial resistance, even at low concentrations (Schwartz et al., 2003; Auerbach et al., 2007; Adelowo et al., 2008; Baquero et al., 2008; Tahrani et al., 2015). Three fundamental mechanisms of antimicrobial resistance could be summarized as follows: enzymatic degradation of antibacterial drugs, alteration of bacterial proteins that are targeted by antimicrobials, and changes in membrane permeability to antibiotics (Dever, 1991).

The dissemination of antimicrobial resistance (AMR) among bacterial communities is one of the biggest challenges faced by mankind in the public health domains. Antibiotic resistance of bacteria is a biological risk, which increases morbidity and mortality of animals and humans (EFSA, 2008). The persistence of antimicrobial-resistant genes in the soil environment is a concern (Yang et al., 2020).

Sequencing of the 16S ribosomal RNA gene has served as an important tool for the determination of phylogenetic relationships between bacteria (Nübel et al., 1996; Hesham, 2014; Hesham et al., 2014a,b; Mawad et al., 2016). Bacteria isolated in the current study were identified as *S. haemolyticus* (AH-03), *E. faecalis* (AH-07) and *E. coli* (AH-13) using the 16S rRNA gene sequence and phylogenetic tree analysis. They were resistant to the five tested antibiotics; therefore, they were considered multi-drug resistant bacteria. The presence of antimicrobial-resistant bacteria has been reported in several studies in polluted and non-polluted environments (Harwood et al., 2000; Martins da Costa et al., 2006; Narciso-da-Rocha and Manaia, 2016; Dungan and Bjorneberg, 2021).

Similar results of high values of resistance to ampicillin and chloramphenicol have been reported for all tested strains of *E. coli* in previous studies (Solomakos et al., 2009; Tahrani et al., 2015; Narciso-da-Rocha and Manaia, 2016; Arefa et al., 2021; Dungan and Bjorneberg, 2021). High values of resistance to chloramphenicol were recorded for *Enterococcus* sp., *Staphylococcus aureus*, and *E. coli* that were isolated from hospitals in the study of Martins da Costa et al. (2006). Isolates belonging to *E. faecalis* and *E. coli* were determined to be Multi-drug resistant to up to six different antimicrobial drug classes (Dungan and Bjorneberg, 2021). These findings came concomitant with the results of the current study where antibiotic resistance was prevalent in the isolated bacteria against the five tested antibiotics that are commonly used.

To date, much effort has been devoted to the control of cell-associated propagation of AMR. However, substantial knowledge gaps remain on the contribution of cell-free DNA to promote horizontal transfers of resistance genes in wastewater and downstream environments (Woegerbauer et al., 2020).

Cell-free DNAs may well represent a significant source for ARG dissemination that suffers a lack of attention although it should be considered a hazard in the context of wastewater reuse. ARGs encoded on free extracellular DNA (exDNA), persist throughout different WWTP compartments (1–16 ng/mL), and ARG-associated exDNA is present in substantial amounts even in purified discharged effluents (Zhang et al., 2018). Natural transformation describes the physiologically regulated uptake and genomic integration of free exDNA by competent bacteria (Magnus Nielsen and van Elsas, 2019).

However, the situation is unclear as fragments of cell-free DNA show long-term persistence (several years) in soil and other natural environments (Overballe-Petersen et al., 2013), and despite adsorption to soil particles, exDNA is capable to transform competent cells in these environments (Thomas and Nielsen, 2005; Morrissey et al., 2015).

Zucchini as an important commercial crop that has gained popularity in both open-field and greenhouses in the Mediterranean region (Rouphael and Colla, 2005) was selected for the greenhouse experiment. In this study, hospital-treated wastewater was reused in the irrigation of zucchini seedlings (compared to freshwater) to check if it will have any good effect on seedlings growth despite the well-considered pollutants that it may contain. Results indicated a little improvement in seedlings grown in the case of hospital effluent irrigated seedlings, than that irrigated with fresh water, as indicated by fresh weights of total seedlings, roots, and leaves. This could be explained by the presence of utilizable organic compounds and some minerals obtained from the degradation of organic matter contained in hospital wastewater during the treatment process. Although numerous studies that examined the reuse of treated wastewater in agriculture reported yield increase, improved soil and crop quality, and reduction in fertilizer dose (Cirelli et al., 2012), the authors highlighted the risk of contaminants such as total coliforms and *E. coli*.

Numerous studies have examined the use of treated wastewater in agriculture for crops including lettuce, tomatoes, and eggplants (Cirelli et al., 2012). In addition to increasing yield, improving soil and crop quality, and reducing fertilizer dose, the authors concluded that contaminants like total *coliforms* and *Escherichia coli* remain a concern. In recent years, scientific research has recommended ways of reducing these undesirable outcomes (Gadow et al., 2023).

Putting back these observations into the water reuse context, special care should be given to the cell-free DNA that harbors ARG (Woegerbauer et al., 2020); therefore, cell-free ARGs might be a kind of important but previously neglected pollutant from WWTPs, which added a possible risk to the hospital effluent receiving environments (Zhang et al., 2018).

Yang et al. (2014) described ARGs that are removed by neither physical, chemical, or biological treatment processes, as being designated as persistent. Different technologies that could help in the removal of ARBs and ARGs from wastewater effluents include membrane bioreactor treatments (Riquelme Breazeal et al., 2013) or algal-based wastewater treatment systems (Cheng et al., 2020) that are nonrenewable and need consistent variations on the WWTP operation processes. The sewage sludge-derived biochar is a promising material that can make significant contributions on adsorption, hence the removal of cell-free DNA and ARGs is a promising method for the management of wastewater (Rangabhashiyam et al., 2022). The effectiveness of coagulation technology in the removal of ARGs from treated wastewater (*via* two coagulants: FeCl3 and polyferric chloride) was investigated and recommended (Li et al., 2017). Targets for wastewater treatment could be achieved through a combination of different technologies to minimize risks (González et al., 2016).

In this study, the Authors recommend the application of *Bacillus subtilis* in the secondary treatment (aeration tank) of a hospital wastewater treatment plant. *B. subtilis* is an aerobic, generally regarded as safe (GRAS) bacterium (Nguyen and Phan, 2022). It is considered a probiotic with remarkable anti-microorganism activity (Wang et al., 2017). Besides the great advantage of its non-toxic nature (Khodavirdipour et al., 2022), *B. subtilis* was reported to have a nuclease activity that could participate in the biodegradation of cell-free DNA and ARGs (Kruszewska et al., 2004). Exonucleases of *B*. *subtilis* degrade DNA to deoxyribonucleoside 3′-monophosphates (Kanamori et al., 1974). *Bacillus* species (including *B. subtilis*) showed the highest biosurfactant activities (Khodavirdipour et al., 2022). The antiviral activity of surfactin, produced by *B. subtilis*, was confirmed for a broad range of viruses especially enveloped ones such as COVID-19, retroviruses, and herpes. Surfactin disrupts the viral lipid membrane and partially affects the capsid as was observed by electron microscopy (Vollenbroich et al., 1997). Fecal-oral transmission of viruses was reported as a potential route of transmission, and the presence of SARS-CoV-2 RNA in the feces of infected patients and in hospital wastewater was confirmed by previous studies. The application of *Bacillus subtilis* in the HWW treatment could facilitate the inactivation of enveloped viruses and the biodegradation of cell-free DNA including ARG. Efficient and updated technologies should be applied to avoid another wave of the pandemic of COVID-19 infections (Achak et al., 2021). Many studies strongly recommend the on-site treatment of HWW to reduce the potential risks (Yan et al., 2020). Leftover hospital contaminations should be carefully considered before allowing water reuse or evacuation to the environment. Based on these findings, the removal of viruses, antibiotic-resistant bacteria, as well as the persistent, long neglected cell-free DNA as a source for ARG from effluent hospital WWTP should be carefully considered for a more safe discharge to the environment.

## 5. Conclusion

Multi-drug resistant bacteria were recovered from the effluent of Beni-Suef university hospital (three isolates). They exhibited resistance to five of the different commonly used antibiotics (Tetracycline, Ampicillin, Amoxicillin, Chloramphenicol, and Erythromycin). Isolates were identified using 16S rRNA gene and sequence homology as *Staphylococcus haemolyticus* (AH-03), *Enterococcus faecalis* (AH-07), and *Escherichia coli* (AH-13). The greenhouse experiment results showed that there were no significant differences in the total zucchini plant fresh weight using freshwater or the treated hospital effluent water. Our results demonstrated the potential of the hospital wastewater effluent in spreading multi-drug resistance either as multi-antibiotic-resistant bacteria or as the long-lasting hazardous cell-free DNA that carries antibiotic resistance genes. Spread of viruses should be also considered to avoid another wave of the pandemic of COVID-19 infections. The benefit of plant growth improvement that could be gained from the reuse of treated hospital wastewater is incomparable to the huge risk to the environment and public health that results from spreading of the antibiotic resistance to soil bacteria and aquatic environments. Removal of viruses, antibiotic-resistant bacteria, as well as cell-free DNA and ARGs, is strongly recommends treatment for waste waters especially those from hospitals before discharge to downstream environments.

## Data availability statement

The datasets presented in this study can be found in online repositories. The names of the repository/repositories and accession number(s) can be found at: https://www.ncbi.nlm.nih.gov/genbank/, ON873252, ON873253, and ON873254.

## Author contributions

MMM, SIG, and AH: designed the study, investigation and writing original draft preparation. FA, YM, KZ, NF and RF: software and review. AH: review and editing. All authors read and approved the final manuscript version.

## Funding

This research was supported by a grant from Academy of Scientific Research and Technology (ASRT), Egypt, through the bilateral research proposals with the National Natural Science Foundation of China (ASRT-NSFC call 2018).

## Conflict of interest

The authors declare that the research was conducted in the absence of any commercial or financial relationships that could be construed as a potential conflict of interest.

## Publisher’s note

All claims expressed in this article are solely those of the authors and do not necessarily represent those of their affiliated organizations, or those of the publisher, the editors and the reviewers. Any product that may be evaluated in this article, or claim that may be made by its manufacturer, is not guaranteed or endorsed by the publisher.

## Footnotes

^1^ https://www.ncbi.nlm.nih.gov/genbank/
